# Age and sex differences in the effects of short- and long-term exposure to air pollution on endothelial dysfunction

**DOI:** 10.1186/s12940-024-01100-3

**Published:** 2024-07-08

**Authors:** Haoyu Zhang, Jing Yang, Yinghua Zhang, Keling Xiao, Yang Wang, Jin Si, Yan Li, Lijie Sun, Jinghao Sun, Ming Yi, Xi Chu, Jing Li

**Affiliations:** 1 Department of Geriatrics, National Clinical Research Center for Geriatric Diseases, Xuanwu Hospital, Capital Medical University, Beijing, 100053 China; 2 Department of Cardiology, Chuiyangliu Hospital Affiliated to Tsinghua University, Beijing, 100021 China; 3 Medical Research & Biometrics Center, National Center for Cardiovascular Diseases, Fuwai Hospital, Chinese Academy of Medical Sciences and Peking Union Medical College, Beijing, 100037 China; 4 Health Management Center, Xuanwu Hospital, Capital Medical University, Beijing, 100053 China

**Keywords:** Air pollution, Endothelial dysfunction, Flow-mediated dilatation

## Abstract

**Background:**

The effects of air pollution on endothelial function remain unclear across populations. We aimed to use brachial artery flow-mediated dilatation (FMD) to identify demographic differences in the effects of air pollution exposure on endothelial dysfunction.

**Methods:**

We measured FMD in 850 participants from October 2016 to January 2020. Location-specific concentrations of fine particulate matter *<* 2.5 μm aerodynamic diameter (PM_2.5_), inhalable particulate matter *<* 10 μm aerodynamic diameter (PM_10_), sulfur dioxide (SO_2_), nitrogen dioxide (NO_2_), carbon monoxide (CO), and ozone (O_3_) measured by fixed ambient air monitoring stations were collected for short- and long-term exposure assessment. Multiple linear regression models and restricted cubic splines were used to assess the associations before and after stratification by age and sex.

**Results:**

This study eventually included 828 participants [551 (66.5%) younger than 65 years and 553 (66.8%) men]. Each 10 µg/m^3^ increase in 7-day exposure to PM_2.5_ and PM_10_ was significantly linearly associated with a 0.07% (*β* = -0.07, 95% CI: -0.13 to -0.004) and 0.05% (*β* = -0.05, 95% CI: -0.10 to -0.004) decrease in FMD in the fully adjusted model. After full adjustment, long-term exposure to all air pollutants was significantly associated with impaired FMD. Each 10 µg/m^3^ increase in long-term exposure to PM_2.5_ and PM_10_ was significantly associated with a -0.18% (95% CI: -0.34 to -0.03) and − 0.23% (95% CI: -0.40 to -0.06) change in FMD, respectively. After stratification, the associations of lower FMD with long-term exposure to PM_2.5_, PM_10_, SO_2_, NO_2_, and CO significantly persisted in men and participants younger than 65 years instead of women or older participants. For short-term exposure, we observed differences consistent with long-term exposure and a stronger effect of 7-day exposure to SO_2_ in men due to a significant interaction effect.

**Conclusion:**

Short- and long-term exposure to different air pollutants are strongly associated with decreased endothelial function, and susceptibility to air pollution varies significantly with age and sex.

**Supplementary Information:**

The online version contains supplementary material available at 10.1186/s12940-024-01100-3.

## Background

Age and sex-related disparities in the primary and secondary prevention of cardiovascular disease (CVD) are a continually pressing public issue. With a rapid and sustained increase in the ageing population, the rising CVD mortality rate among older patients presents a great challenge to the disease burden in China [1, 2]. Besides, it is suggested that women with prior CVD are less likely to take guideline-directed medications, and those with a high risk of CVD are less likely to have well-controlled blood pressure, low-density lipoprotein cholesterol (LDL-C), or body mass index than men [3]. Since ageing and sex are irreversible, it is essential to improve public health policies and protect the aforementioned populations from other CVD risk factors.

As a global concern and an ever-increasing burden on national health systems [4, 5], air pollution exposure has been uncovered to increase CVD morbidity and mortality in healthy populations [6, 7], and to aggravate the prognoses of those with pre-existing CVD [8, 9]. The vascular endothelium is a biologically active tissue that plays a central role in the process of atherosclerosis, from early to advanced stages, by regulating vascular tone, modulating vascular inflammation, thrombosis, and vascular injury mainly via radical nitric oxide [10]. Endothelial dysfunction is therefore an integrated index of risk factor burden and a marker of subclinical atherosclerotic cardiovascular disease (ASCVD). Brachial flow-mediated dilation (FMD) is a widely used noninvasive test of peripheral endothelial function. Impaired FMD has been demonstrated to predict adverse cardiovascular events and worse cardiovascular outcomes, showing great value in primary and secondary prevention strategies for CVD [11, 12]. However, epidemiological studies on the association between air pollution and endothelial dysfunction provide conflicting evidence and focus almost exclusively on the effects of lower and narrower ranges of particulate matter on healthy populations [13–16]. Consequently, differences in susceptibility by age and sex have not been adequately elucidated in the current studies on endothelial dysfunction.

In this study, we hypothesized that exposure to air pollution would be consistently and positively associated with persistent endothelial dysfunction, and more severe effects were expected to be observed in female and older participants. We explored the impacts of six air pollutants across different time windows in a more heavily polluted area and their differences across populations to test the hypothesis. We aimed to provide new insights into the prevention of CVD by assessing endothelial dysfunction to identify vulnerable populations under air pollution exposure.

## Patients and methods

### Study population

This study was carried out at Xuanwu Hospital, Capital Medical University from October 2016 to January 2020. The study participants were enrolled from: (1) patients with a known history of CVD who came for routine visits to the Cardiology Outpatient Department; and (2) subjects who attended regular physical examinations at the Health Management Department. Those who volunteered for additional cardiovascular risk assessments and endothelial function measurements at the Health Management Department were enrolled in this study consecutively. End-stage liver or kidney disease, incomplete primary data, and residence outside Xicheng District, Beijing were the main exclusion criteria. The study was conducted following the Declaration of Helsinki and approved by the Ethics Committee of Xuanwu Hospital, Capital Medical University. Written informed consent was received from all participants enrolled in the study.

Age, sex, body mass index (BMI), smoking status (never smoker, former smoker, and current smoker), physical examination (resting heart rate, systolic and diastolic blood pressure), laboratory tests (triglycerides, high-density lipoprotein cholesterol, LDL-C, serum uric acid, and serum creatinine), treatment medication, comorbidities (CVD, hypertension, diabetes, and other medical diagnosis), and residential address were obtained through available medical records and standardized questionnaires by trained interviewers. The CVD history of all participants was diagnosed based on coronary angiography or coronary computed tomography or a history of myocardial infarction; other diagnoses were based on self-reported, previous medical history, and available physical examination medical records. The Prediction for ASCVD Risk in China (China-PAR) model, one of the extra cardiovascular risk assessments, was used to predict the 10-year ASCVD risk to characterize the population of non-ASCVD participants in this study (e.g., < 5.0% as low risk, 5.0-9.9% as moderate risk, and ≥ 10.0% as high risk) [17].

### Measures of endothelial dysfunction

Endothelial dysfunction was measured by FMD according to the standardized procedures [18, 19]. FMD was detected by the same ultrasound researcher, and the brachial artery was scanned with a color Doppler ultrasound instrument. All participants fasted (> 6 h), avoided exercise (> 12 h), and abstained from vitamin C, caffeine, polyphenols, supplements, alcohol, and smoking (> 12 h). Participants were placed in the supine position and the brachial artery of the right arm was selected for examination after 15 min of rest. The sphygmomanometer cuff was tied 1–2 cm distal to the antecubital fossa. The baseline artery diameter (BAD) was scanned and examined longitudinally above the antecubital fossa for more than 30 s before the cuff inflation. The cuff pressure was then 50 mmHg higher than systolic pressure (1 mmHg = 0.133 kPa) and inflated for 5 min to occlude blood flow. The pressure in the cuff was monitored within 5 min to ensure that the pressure fluctuation was less than 10 mmHg. After cuff deflation, the diameter was measured continuously for 3 min to obtain the maximum artery diameter (MAD). All diameter measurements were obtained at the end diastole.

FMD (%) was computed as: (MAD-BAD/BAD) ×100.

### Exposure assessment

Air pollutant data were obtained from the National Air Pollution Monitoring System, including fine particulate matter with aerodynamic diameter < 2.5 μm (PM_2.5_), inhalable particulate matter with aerodynamic diameter < 10 μm (PM_10_), sulfur dioxide (SO_2_), nitrogen dioxide (NO_2_), carbon monoxide (CO), and ozone (O_3_). Xicheng District is a relatively small administrative area in the center of Beijing, with a width of about 7.1 km from east to west and a length of about 11.2 km from north to south, a total area of 50.70 km^2^ and two fixed air quality monitoring stations. Daily (24-hour) average concentrations of PM_2.5_, PM_10_, NO_2_, SO_2_, CO, and 8-hour maximum ozone levels were the average values measured by the two fixed ambient air quality monitoring stations in Xicheng District, Beijing. The above pollutant concentrations were used directly in the exposure assessments for all participants, considering the proximity of the addresses to the monitoring stations, as all participants lived in Xicheng District. Daily meteorological data on temperature (℃) were also obtained from the China Meteorological Administration.

We used the moving average 7- and 14-day exposures before the date of FMD measurements to estimate the short-term exposure effects and the moving average 12-month exposures before the date of FMD measurements to estimate the long-term exposure effects. Corresponding time windows (7- and 14-day) were also assigned to temperature.

### Statistical analysis

Baseline characteristics of participants were described as mean ± standard deviation (SD) for normally distributed continuous variables, median (25th, 75th percentiles) for variables with skewed distributions, and numbers (percentages) for categorical variables. The Shapiro–Wilk normality test was performed to test the normality of the data. Continuous variables were compared using the Mann-Whitney’s *U* test and proportions for categorical variables were compared using the chi-square test or the Fisher exact test. Spearman correlation analysis was used to calculate associations between different pollutant concentrations at different exposure times.

First, we grouped air pollution concentration quartiles (Quartiles 1 to 4) and performed crude analyses to assess the association between air pollutants and FMD for different exposure time windows. Multiple linear regression models were then performed to examine the associations further and calculate the partial regression coefficient (*β*) and its 95% confidence interval (95% CI). Baseline variables that were considered clinically relevant or with *P* values of less than 0.10 in univariate analysis were entered into the models while avoiding multicollinearity and over-adjustment. For short-term exposure, Model 1 adjusted for age group (< 65 years and ≥ 65 years), sex, BMI, smoking status, temperature, and season (warm: April to September; cold: October to March) as the covariates. “Season” was added to control for possible seasonal trends in short-term exposure effects. For long-term exposure, Model 1 adjusted for the same covariates except temperature and season. Model 2 additionally adjusted for CVD, hypertension, diabetes mellitus, resting heart rate, and LDL-C. We used the results from Model 2 as the final results. The median concentrations of each quartile group of air pollutants were entered into the models as continuous variables to test for linear trends. Restricted cubic splines with three knots were used to verify and visualize the statistically significant associations identified by regression analysis. To determine whether the associations between air pollutants and FMD were consistent across demographic subgroups, we tested for interaction by age and sex and reconstructed stratified fully adjusted analyses.

In the sensitivity analyses, we reran the model with all air pollutant concentrations as continuous variables to determine the corresponding FMD change values for each 10 µg/m^3^ increase in PM_2.5_, PM_10_, NO_2_, and O_3_, each 1 µg/m^3^ increase in SO_2_, and each 0.1 mg/m^3^ increase in CO. Then, we added the year of observation as a covariate in Model 1 to adjust for possible time trends. Besides, we added both long-term and short-term exposure to Model 1 to assess whether both effects persist when combined. Finally, we constructed two-pollutant models based on Model 1 to assess the robustness of each pollutant effect. Because long-term exposure concentrations of air pollutants other than ozone were strongly correlated (Spearman’s correlation coefficient > 0.9), we did not include them in the same model to avoid potential collinearity.

Two-sided *P* values < 0.05 were considered statistically significant. All statistical analyses were performed with SPSS version 26.0 (Inc., Chicago, IL, USA) and R version 4.3.2 (R Foundation for Statistical Computing, Vienna, Austria).

## Results

A total of 850 participants underwent endothelial function measurements during the study period, with 828 participants eventually included in the study. Two participants were excluded due to end-stage renal disease and 20 participants were excluded due to incomplete baseline data or non-Xicheng District addresses. 33.5% of participants were aged 65 years or older and 66.8% were male. The predicted 10-year ASCVD risks for participants without CVD using the China-PAR model were 6.7 (3.2, 11.5) %. Baseline characteristics of study participants are shown in Table 1. Air pollutant exposure levels for all participants are summarized in the Additional file: Table S1. The map of Beijing labelled with the two fixed air monitoring stations in Xicheng District is shown in the Additional file: Figure S1. Correlations between air pollutants for three different exposure times are presented in the Additional file: Table S2, Table S3, and Table S4. The correlations between short-term exposure concentrations of gaseous pollutants were moderate, whereas there were higher correlations between particulate matter since PM_2.5_ is the large fraction of PM_10_. Except for ozone, the long-term exposure levels for the other five air pollutants were strongly correlated (Spearman’s correlation coefficients > 0.9).

**Table 1:** Baseline characteristics of the study participants^a, b^

Characteristic	Participants (*n* = 828)	Age			Sex		
		< 65 years (*n* = 551)	≥ 65 years (*n* = 277)	*P* value	Male (*n* = 553)	Female (*n* = 275)	*P* value
Age ≥ 65 years, n (%)	277 (33.5)	——	——	——	172 (31.1)	105 (38.2)	0.042
Male, n (%)	553 (66.8)	381 (69.1)	172 (62.1)	0.042	——	——	——
BMI, kg/m^2^	25.4 (23.2, 27.8)	25.8 (23.4, 28.1)	25.0 (22.8, 27.2)	0.004	25.8 (23.6, 28.1)	24.9 (22.5, 27.2)	< 0.001
Smoking status, n (%)				< 0.001			< 0.001
Never	430 (51.9)	268 (48.6)	162 (58.5)		174 (31.5)	256 (93.1)	
Former	142 (17.1)	77 (14.0)	65 (23.5)		135 (24.4)	7 (2.5)	
Current	256 (30.9)	206 (37.4)	50 (18.1)		244 (44.1)	12 (4.4)	
CVD, n (%)	511 (61.7)	298 (54.1)	213 (76.9)	< 0.001	374 (67.6)	137 (49.8)	< 0.001
Hypertension, n (%)	502 (60.6)	297 (53.9)	205 (74.0)	< 0.001	331 (59.9)	171 (62.2)	0.519
Diabetes mellitus, n (%)	246 (29.7)	138 (25.0)	108 (39.0)	< 0.001	171 (30.9)	75 (27.3)	0.279
Physical examination							
Heart rate, beats/min	70 (64, 77)	70 (64, 78)	69 (62, 77)	0.106	70 (64, 78)	69 (63, 76)	0.405
SBP, mmHg	132 (120, 145)	130 (119, 142)	135 (122, 150)	0.001	131 (120, 144)	132 (119, 148)	0.367
DBP, mmHg	77 (69, 86)	79 (70, 86)	75 (66, 81)	< 0.001	80 (71, 87)	73 (66, 80)	< 0.001
Laboratory test							
Triglycerides, mmol/L	1.5 (1.1, 2.1)	1.6 (1.1, 2.3)	1.4 (1.0, 1.9)	< 0.001	1.5 (1.1, 2.2)	1.4 (1.1, 2.1)	0.258
LDL-C, mmol/L	2.5 (2.0, 3.1)	2.6 (2.0, 3.2)	2.3 (1.8, 2.8)	< 0.001	2.4 (1.9, 3.0)	2.6 (2.0, 3.2)	0.051
HDL-C, mmol/L	1.1 (0.9, 1.3)	1.1 (0.9, 1.4)	1.1 (0.9, 1.3)	0.688	1.1 (0.9, 1.2)	1.3 (1.0, 1.6)	< 0.001
Serum uric acid, µmol/L	341.5 (284.0, 400.8)	347.0 (285.0, 409.0)	331.0 (278.5, 384.0)	0.010	365.0 (310.0, 421.0)	290.0 (241.0, 347.0)	< 0.001
Serum creatinine, µmol/L	68.0 (57.0, 77.0)	67.0 (57.0, 77.0)	69.0 (57.0, 79.0)	0.380	72.0 (65.0, 81.0)	54.0 (48.0, 63.0)	< 0.001
FMD, %	5.1 (3.4, 7.1)	5.4 (3.5, 7.7)	4.8 (3.2, 6.3)	< 0.001	5.0 (3.2, 6.9)	5.5 (3.8, 7.7)	0.006

^a^ BMI, body mass index; CVD, cardiovascular disease; SBP, systolic blood pressure; DBP, diastolic blood pressure; LDL-C, low-density lipoprotein cholesterol; HDL-C, high-density lipoprotein cholesterol; FMD, flow-mediated dilation

^b^ Values are mean (standard deviation) or median (25th, 75th percentiles) for continuous variables and percentage for categorical variables

As shown in Table 2, each 10 µg/m^3^ increase in 7-day exposure to PM_2.5_ and PM_10_ was significantly linearly associated with a 0.07% (*β* = -0.07, 95% CI: -0.13 to -0.004) and 0.05% (*β* = -0.05, 95% CI: -0.10 to -0.004) decrease in FMD in the fully adjusted model. Although per 10 µg/m^3^ increase in O_3_ was significantly associated with a -0.08% change in FMD (*β* = -0.08, 95% CI: -0.15 to -0.005), there is an obvious nonlinear relationship between O_3_ concentration and FMD (*P*_trend_ = 0.096). For 14-day exposure, we observed a trend for ozone to be associated with endothelial dysfunction after full adjustment (*β* = -0.08, 95% CI: -0.17 to 0.004; *P* = 0.063).

**Table 2:** Associations between short-term exposure to air pollution and flow-mediated dilation (%) among all participants^a, b^

			Quartile 2^*^		Quartile 3^*^		Quartile 4^*^		*P*_trend_	Continuous variables^**^	
			β (95% CI)	*P* value	β (95% CI)	*P* value	β (95% CI)	*P* value		β (95% CI)	*P* value
7-day	PM_2.5_	Crude model	-0.13 (-0.68, 0.41)	0.632	-0.19 (-0.74, 0.35)	0.490	-0.42 (-0.97, 0.13)	0.131	0.125	-0.05 (-0.11, 0.02)	0.177
		Model 1	-0.08 (-0.61, 0.46)	0.777	-0.10 (-0.64, 0.44)	0.723	-0.42 (-0.97, 0.13)	0.138	0.124	-0.05 (-0.11, 0.02)	0.155
		Model 2	-0.08 (-0.59, 0.43)	0.756	-0.13 (-0.65, 0.39)	0.620	-0.55 (-1.08, -0.02)	0.043	0.033	-0.07 (-0.13, -0.004)	0.037
	PM_10_	Crude model	-0.19 (-0.74, 0.35)	0.486	0.06 (-0.48, 0.61)	0.821	-0.54 (-1.08, 0.004)	0.052	0.067	-0.05 (-0.10, 0.006)	0.086
		Model 1	-0.06 (-0.60, 0.47)	0.817	0.15 (-0.40, 0.70)	0.594	-0.41 (-0.96, 0.14)	0.139	0.139	-0.04 (-0.09, 0.01)	0.120
		Model 2	-0.17 (-0.69, 0.35)	0.515	0.10 (-0.43, 0.63)	0.715	-0.60 (-1.13, -0.07)	0.027	0.032	-0.05 (-0.10, -0.004)	0.036
	SO_2_	Crude model	-0.06 (-0.60, 0.47)	0.814	0.11 (-0.44, 0.66)	0.690	-0.56 (-1.11, -0.02)	0.042	0.026	-0.02 (-0.06, 0.04)	0.557
		Model 1	-0.09 (-0.64, 0.45)	0.738	0.07 (-0.55, 0.68)	0.833	-0.60 (-1.32, 0.12)	0.103	0.053	-0.004 (-0.06, 0.06)	0.887
		Model 2	-0.22 (-0.74, 0.31)	0.420	-0.08 (-0.68, 0.51)	0.790	-0.78 (-1.47, -0.08)	0.030	0.019	-0.04 (-0.09, 0.02)	0.222
	NO_2_	Crude model	-0.34 (-0.88, 0.20)	0.218	-0.48 (-1.03, 0.07)	0.087	-0.13 (-0.67, 0.42)	0.647	0.727	-0.01 (-0.15, 0.12)	0.839
		Model 1	-0.17 (-0.72, 0.38)	0.537	-0.34 (-0.96, 0.28)	0.279	-0.07 (-0.75, 0.61)	0.843	0.930	0.003 (-0.16, 0.17)	0.973
		Model 2	-0.25 (-0.77, 0.28)	0.362	-0.43 (-1.02, 0.17)	0.163	-0.25 (-0.90, 0.41)	0.459	0.540	-0.06 (-0.22, 0.11)	0.492
	CO	Crude model	0.59 (0.04, 1.13)	0.035	-0.21 (-0.74, 0.32)	0.430	-0.22 (-0.76, 0.32)	0.429	0.105	-0.01 (-0.05, 0.03)	0.578
		Model 1	0.53 (-0.007, 1.07)	0.053	-0.24 (-0.77, 0.29)	0.369	-0.24 (-0.82, 0.35)	0.421	0.111	-0.01 (-0.06, 0.03)	0.608
		Model 2	0.50 (-0.02, 1.03)	0.058	-0.15 (-0.66, 0.36)	0.576	-0.45 (-1.01, 0.12)	0.123	0.024	-0.03 (-0.07, 0.01)	0.189
	O_3_	Crude model	-0.46 (-1.01, 0.09)	0.098	-0.39 (-0.93, 0.16)	0.162	-0.23 (-0.78, 0.32)	0.410	0.618	-0.009 (-0.05, 0.03)	0.634
		Model 1	-0.55 (-1.10, -0.005)	0.048	-1.20 (-2.16, -0.24)	0.015	-1.27 (-2.36, -0.19)	0.022	0.053	-0.08 (-0.15, -0.005)	0.037
		Model 2	-0.37 (-0.90, 0.16)	0.170	-0.98 (-1.91, -0.05)	0.040	-1.06 (-2.12, -0.01)	0.048	0.096	-0.08 (-0.15, -0.005)	0.036
14-day	PM_2.5_	Crude model	-0.31 (-0.86, 0.23)	0.261	-0.43 (-0.97, 0.12)	0.127	-0.25 (-0.79, 0.30)	0.378	0.493	-0.002 (-0.08, 0.08)	0.971
		Model 1	-0.21 (-0.75, 0.33)	0.446	-0.33 (-0.88, 0.21)	0.226	-0.18 (-0.73, 0.37)	0.527	0.596	0.004 (-0.08, 0.09)	0.919
		Model 2	-0.25 (-0.77, 0.27)	0.350	-0.44 (-0.96, 0.09)	0.101	-0.30 (-0.84, 0.23)	0.264	0.306	-0.04 (-0.12, 0.05)	0.402
	PM_10_	Crude model	-0.33 (-0.88, 0.21)	0.231	-0.34 (-0.89, 0.21)	0.220	-0.30 (-0.85, 0.24)	0.275	0.367	-0.03 (-0.09, 0.03)	0.353
		Model 1	-0.25 (-0.79, 0.30)	0.374	-0.21 (-0.78, 0.36)	0.469	-0.15 (-0.72, 0.42)	0.611	0.756	-0.01 (-0.08, 0.05)	0.675
		Model 2	-0.31 (-0.83, 0.22)	0.256	-0.21 (-0.76, 0.34)	0.446	-0.31 (-0.86, 0.24)	0.271	0.393	-0.04 (-0.10, 0.03)	0.256
	SO_2_	Crude model	-0.06 (-0.60, 0.48)	0.817	-0.46 (-1.01, 0.09)	0.099	-0.47 (-1.02, 0.07)	0.090	0.062	-0.02 (-0.07, 0.03)	0.452
		Model 1	-0.08 (-0.64, 0.47)	0.774	-0.51 (-1.15, 0.13)	0.120	-0.50 (-1.31, 0.31)	0.224	0.255	0.005 (-0.06, 0.07)	0.881
		Model 2	-0.06 (-0.60, 0.47)	0.822	-0.56 (-1.18, 0.06)	0.078	-0.63 (-1.41, 0.15)	0.114	0.115	-0.03 (-0.09, 0.04)	0.411
	NO_2_	Crude model	-0.37 (-0.92, 0.17)	0.180	-0.64 (-1.18, -0.10)	0.021	-0.03 (-0.57, 0.52)	0.915	0.768	0.02 (-0.14, 0.18)	0.793
		Model 1	-0.18 (-0.73, 0.37)	0.522	-0.37 (-1.04, 0.29)	0.268	0.09 (-0.63, 0.81)	0.812	0.782	0.09 (-0.11, 0.30)	0.376
		Model 2	-0.28 (-0.81, 0.25)	0.293	-0.48 (-1.12, 0.16)	0.139	-0.02 (-0.72, 0.67)	0.948	0.993	0.02 (-0.19, 0.22)	0.884
	CO	Crude model	-0.27 (-0.81, 0.28)	0.339	-0.56 (-1.10, -0.01)	0.045	-0.41 (-0.95, 0.14)	0.145	0.151	0.02 (-0.03, 0.07)	0.489
		Model 1	-0.31 (-0.86, 0.25)	0.285	-0.50 (-1.06, 0.06)	0.077	-0.39 (-1.01, 0.22)	0.205	0.247	0.03 (-0.03, 0.08)	0.326
		Model 2	-0.31 (-0.85, 0.23)	0.257	-0.37 (-0.91, 0.17)	0.180	-0.47 (-1.06, 0.12)	0.119	0.149	0.003 (-0.05, 0.06)	0.927
	O_3_	Crude model	-0.18 (-0.72, 0.37)	0.529	0.12 (-0.43, 0.67)	0.662	-0.16 (-0.71, 0.39)	0.568	0.872	-0.004 (-0.04, 0.04)	0.846
		Model 1	-0.33 (-0.90, 0.23)	0.251	-0.18 (-1.55, 1.20)	0.800	-0.60 (-2.07, 0.88)	0.428	0.119	-0.09 (-0.18, -0.001)	0.047
		Model 2	-0.20 (-0.75, 0.35)	0.476	-0.11 (-1.43, 1.22)	0.877	-0.46 (-1.89, 0.96)	0.522	0.204	-0.08 (-0.17, 0.004)	0.063

^a^ PM_2.5_, fine particulate matter with aerodynamic diameter < 2.5 μm; PM_10_, inhalable particulate matter with aerodynamic diameter < 10 μm; SO_2_, sulfur dioxide; NO_2_, nitrogen dioxide; CO, carbon monoxide; O_3_, ozone; 95% CI, 95% confidence interval

^b^ Model 1 adjusted for age group (< 65 years and ≥ 65 years), sex, body mass index, smoking status, temperature, and season (warm: April to September; cold: October to March). Model 2 additionally adjusted for cardiovascular disease, hypertension, diabetes mellitus, resting heart rate, and low-density lipoprotein cholesterol

^*^ Quartile 1 was as the reference

^**^ Continuous variables: per 10 µg/m^3^ increase in PM_2.5_, PM_10_, NO_2_ and O_3_; per 1 µg/m^3^ increase in SO_2_; per 0.1 mg/m^3^ increase in CO

The associations between long-term exposure to air pollution and FMD in all participants are presented in Table 3. Not all concentration quartile subgroups of 12-month air pollution exposure were continuously associated with lower FMD in the different models, especially at lower concentrations (Quartile 2). After full adjustment, each 10 µg/m^3^ increase in long-term exposure to PM_2.5_, PM_10_, NO_2_, and O_3_ was significantly associated with a -0.18% (95% CI: -0.34 to -0.03), -0.23% (95% CI: -0.40 to -0.06), -0.65% (95% CI: -1.05 to -0.25), and − 0.78% (95% CI: -1.57, -0.00) change in FMD, respectively. Long-term exposure to SO_2_ and CO was similarly significantly associated with endothelial dysfunction. Besides, long-term exposure to all pollutants showed significant linear relationships with lower FMD in the tests for linear trends after full adjustment.

**Table 3:** Associations between long-term exposure to air pollution and flow-mediated dilation (%) among all participants^a, b^

12-month exposure		Quartile 2^*^		Quartile 3^*^		Quartile 4^*^		*P*_trend_	Continuous variables^**^	
		β (95% CI)	*P* value	β (95% CI)	*P* value	β (95% CI)	*P* value		β (95% CI)	*P* value
PM_2.5_	Crude model	-0.12 (-0.67, 0.42)	0.657	-0.71 (-1.25, -0.17)	0.010	-0.45 (-0.99, 0.10)	0.109	0.208	-0.11 (-0.27, 0.05)	0.187
	Model 1	-0.15 (-0.68, 0.39)	0.590	-0.61 (-1.14, -0.07)	0.026	-0.45 (-0.99, 0.09)	0.099	0.180	-0.11 (-0.27, 0.05)	0.168
	Model 2	-0.31 (-0.82, 0.21)	0.245	-0.59 (-1.10, -0.07)	0.026	-0.69 (-1.21, -0.17)	0.010	0.025	-0.18 (-0.34, -0.03)	0.020
PM_10_	Crude model	-0.08 (-0.62, 0.47)	0.786	-0.79 (-1.34, -0.24)	0.005	-0.46 (-1.00, 0.09)	0.101	0.097	-0.17 (-0.34, 0.009)	0.063
	Model 1	-0.09 (-0.63, 0.44)	0.737	-0.70 (-1.24, -0.16)	0.011	-0.45 (-0.99, 0.09)	0.099	0.097	-0.16 (-0.33, 0.01)	0.068
	Model 2	-0.28 (-0.80, 0.24)	0.287	-0.65 (-1.17, -0.13)	0.014	-0.69 (-1.21, -0.17)	0.010	0.013	-0.23 (-0.40, -0.06)	0.008
SO_2_	Crude model	-0.15 (-0.69, 0.39)	0.576	-0.72 (-1.27, -0.17)	0.010	-0.45 (-1.00, 0.09)	0.105	0.307	-0.07 (-0.17, 0.03)	0.165
	Model 1	-0.16 (-0.69, 0.37)	0.556	-0.63 (-1.17, -0.09)	0.023	-0.46 (-0.99, 0.08)	0.096	0.253	-0.07 (-0.17, 0.02)	0.130
	Model 2	-0.24 (-0.76, 0.27)	0.354	-0.70 (-1.22, -0.18)	0.009	-0.70 (-1.22, -0.17)	0.009	0.037	-0.13 (-0.22, -0.03)	0.008
NO_2_	Crude model	-0.04 (-0.58, 0.51)	0.896	-0.71 (-1.25, -0.16)	0.011	-0.48 (-1.03, 0.06)	0.082	0.088	-0.53 (-0.95, -0.11)	0.013
	Model 1	0.005 (-0.53, 0.54)	0.984	-0.58 (-1.12, -0.05)	0.033	-0.45 (-0.99, 0.08)	0.098	0.085	-0.51 (-0.92, -0.10)	0.015
	Model 2	-0.09 (-0.62, 0.43)	0.725	-0.54 (-1.06, -0.03)	0.040	-0.67 (-1.20, -0.15)	0.011	0.009	-0.65 (-1.05, -0.25)	0.001
CO	Crude model	-0.26 (-0.81, 0.28)	0.347	-0.60 (-1.14, -0.06)	0.031	-0.44 (-0.99, 0.11)	0.114	0.266	-0.07 (-0.16, 0.03)	0.161
	Model 1	-0.25 (-0.79, 0.29)	0.362	-0.54 (-1.07, -0.001)	0.049	-0.44 (-0.98, 0.10)	0.107	0.232	-0.07 (-0.16, 0.02)	0.142
	Model 2	-0.40 (-0.92, 0.12)	0.128	-0.54 (-1.05, -0.02)	0.042	-0.68 (-1.21, -0.16)	0.011	0.038	-0.11 (-0.20, -0.02)	0.016
O_3_	Crude model	0.001 (-0.54, 0.55)	0.996	0.16 (-0.39, 0.70)	0.570	-0.62 (-1.16, -0.08)	0.026	0.040	-0.91 (-1.72, -0.09)	0.029
	Model 1	-0.04 (-0.58, 0.49)	0.880	0.14 (-0.40, 0.68)	0.609	-0.53 (-1.07, 0.006)	0.053	0.090	-0.77 (-1.58, 0.03)	0.059
	Model 2	-0.09 (-0.61, 0.42)	0.720	0.02 (-0.50, 0.55)	0.929	-0.59 (-1.11, -0.07)	0.027	0.045	-0.78 (-1.57, -0.00)	0.050

^a^ PM_2.5_, fine particulate matter with aerodynamic diameter < 2.5 μm; PM_10_, inhalable particulate matter with aerodynamic diameter < 10 μm; SO_2_, sulfur dioxide; NO_2_, nitrogen dioxide; CO, carbon monoxide; O_3_, ozone; 95% CI, 95% confidence interval

^b^ Model 1 adjusted for age group (< 65 years and ≥ 65 years), sex, body mass index, and smoking status. Model 2 additionally adjusted for cardiovascular disease, hypertension, diabetes mellitus, resting heart rate, and low-density lipoprotein cholesterol

^*^ Quartile 1 was as the reference

^**^ Continuous variables: per 10 µg/m^3^ increase in PM_2.5_, PM_10_, NO_2_ and O_3_; per 1 µg/m^3^ increase in SO_2_; per 0.1 mg/m^3^ increase in CO

**Fig. 1:**
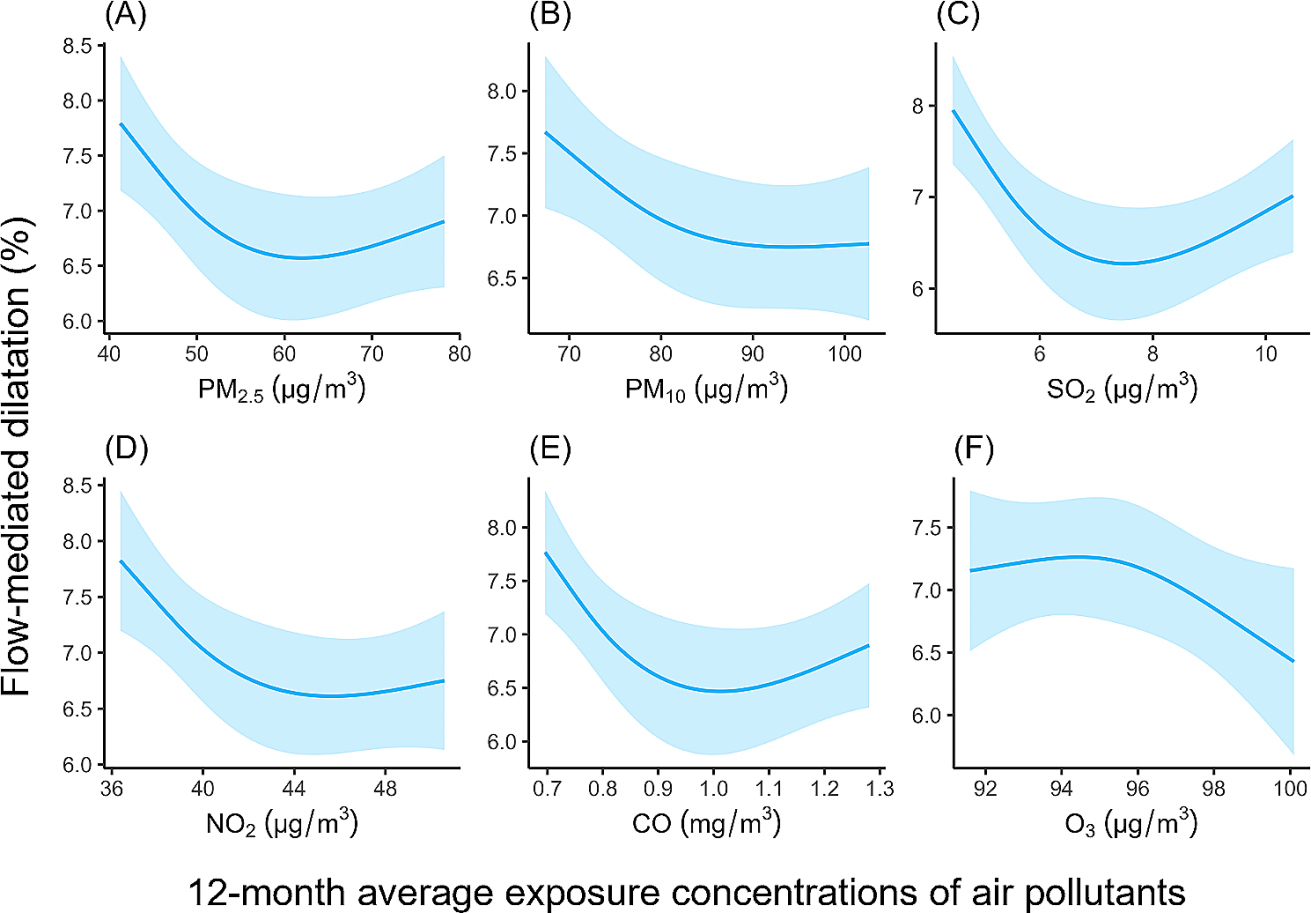
Associations between long-term exposure to (**A**) PM_2.5_, (**B**) PM_10_, (**C**) SO_2_, (**D**) NO_2_, (**E**) CO, (**F**) O_3_ and flow-mediated dilation (%).^a, b^ *Note*: ^a^ PM_2.5_, fine particulate matter with aerodynamic diameter < 2.5 μm; PM_10_, inhalable particulate matter with aerodynamic diameter < 10 μm; SO_2_, sulfur dioxide; NO_2_, nitrogen dioxide; CO, carbon monoxide; O_3_, ozone; 95% CI, 95% confidence interval. ^b^ Model adjusted for age group (< 65 years and ≥ 65 years), sex, body mass index, smoking status, cardiovascular disease, hypertension, diabetes mellitus, resting heart rate, and low-density lipoprotein cholesterol

Table 4 presents the associations between air pollutants and FMD after stratification by sex and age in the fully adjusted models. Significant associations between reduced FMD and exposure to PM_2.5_, PM_10,_ and SO_2_ over 7 days were observed in male participants. Only a notable interaction effect was found between sex and SO_2_ (*P* for interaction = 0.036). The association between 7-day exposure to PM_2.5_ and PM_10_ and endothelial dysfunction was significant in those aged less than 65 years. In addition, 7-day exposure to ozone also tended to be associated with endothelial dysfunction in men and in participants younger than 65 years of age. For long-term exposure, the associations of lower FMD with PM_2.5_, PM_10_, SO_2_, NO_2_, and CO significantly persisted in men and participants younger than 65 years instead of women or older participants. Figure 1 shows refitted concentration-response curves for the associations between 12-month average air pollution exposure concentrations and FMD, which presents associations more directly.

**Table 4:** Associations between exposure to air pollution and flow-mediated dilation (%) after stratification by age and sex^a, b^

	Stratified by	7-day		14-day		12-month	
		β (95% CI)	*P* value	β (95% CI)	*P* value	β (95% CI)	*P* value
PM_2.5_, per 10 µg/m^3^	*P* for interaction		0.144		0.482		0.140
	< 65 years	-0.10 (-0.18, -0.01)	0.028	-0.04 (-0.14, 0.07)	0.476	-0.28 (-0.47, -0.08)	0.006
	≥ 65 years	-0.02 (-0.12, 0.07)	0.653	-0.01 (-0.15, 0.12)	0.855	0.03 (-0.21, 0.28)	0.800
	*P* for interaction		0.186		0.294		0.101
	Male	-0.09 (-0.16, -0.01)	0.021	-0.05 (-0.15, 0.04)	0.285	-0.27 (-0.45, -0.09)	0.004
	Female	-0.03 (-0.17, 0.11)	0.651	-0.01 (-0.18, 0.15)	0.868	-0.03 (-0.32, 0.26)	0.858
PM_10_, per 10 µg/m^3^	*P* for interaction		0.138		0.418		0.184
	< 65 years	-0.08 (-0.14, -0.01)	0.018	-0.05 (-0.13, 0.03)	0.240	-0.32 (-0.54, -0.10)	0.004
	≥ 65 years	-0.009 (-0.09, 0.07)	0.824	-0.007 (-0.11, 0.10)	0.894	-0.02 (-0.29, 0.24)	0.861
	*P* for interaction		0.274		0.330		0.205
	Male	-0.07 (-0.13, -0.01)	0.016	-0.06 (-0.14, 0.01)	0.111	-0.31 (-0.51, -0.11)	0.003
	Female	-0.02 (-0.12, 0.08)	0.655	-0.003 (-0.13, 0.12)	0.965	-0.10 (-0.42, 0.22)	0.526
SO_2_, per 1 µg/m^3^	*P* for interaction		0.115		0.190		0.148
	< 65 years	-0.05 (-0.12, 0.02)	0.126	-0.04 (-0.13, 0.04)	0.290	-0.18 (-0.30, -0.06)	0.003
	≥ 65 years	0.04 (-0.06, 0.15)	0.415	0.02 (-0.08, 0.13)	0.658	0.011 (-0.14, 0.16)	0.886
	*P* for interaction		0.036		0.078		0.055
	Male	-0.07 (-0.13, -0.005)	0.034	-0.06 (-0.13, 0.01)	0.099	-0.19 (-0.30, -0.08)	< 0.001
	Female	0.07 (-0.05, 0.20)	0.252	0.08 (-0.07, 0.23)	0.275	-0.02 (-0.19, 0.16)	0.862
NO_2_, per 10 µg/m^3^	*P* for interaction		0.194		0.157		0.161
	< 65 years	-0.08 (-0.29, 0.14)	0.482	-0.003 (-0.26, 0.26)	0.984	-0.88 (-1.40, -0.36)	< 0.001
	≥ 65 years	-0.01 (-0.25, 0.22)	0.909	0.06 (-0.25, 0.37)	0.703	-0.16 (-0.78, 0.47)	0.620
	*P* for interaction		0.072		0.272		0.174
	Male	-0.11 (-0.30, 0.08)	0.251	0.002 (-0.24, 0.24)	0.985	-0.84 (-1.31, -0.37)	< 0.001
	Female	0.07 (-0.25, 0.39)	0.684	0.004 (-0.37, 0.38)	0.985	-0.33 (-1.10, 0.45)	0.412
CO, per 0.1 mg/m^3^	*P* for interaction		0.463		0.779		0.151
	< 65 years	-0.03 (-0.09, 0.03)	0.267	0.02 (-0.05, 0.09)	0.521	-0.17 (-0.28, -0.05)	0.005
	≥ 65 years	-0.02 (-0.08, 0.04)	0.549	-0.04 (-0.13, 0.05)	0.423	0.01 (-0.13, 0.16)	0.858
	*P* for interaction		0.081		0.074		0.091
	Male	-0.04 (-0.09, 0.006)	0.088	-0.02 (-0.08, 0.04)	0.537	-0.17 (-0.28, -0.06)	0.003
	Female	0.02 (-0.09, 0.14)	0.680	0.05 (-0.06, 0.16)	0.355	-0.02 (-0.19, 0.15)	0.836
O_3_, per 10 µg/m^3^	*P* for interaction		0.151		0.178		0.866
	< 65 years	-0.09 (-0.18, 0.005)	0.063	-0.10 (-0.21, 0.008)	0.069	-0.75 (-1.77, 0.28)	0.153
	≥ 65 years	-0.06 (-0.17, 0.05)	0.269	-0.04 (-0.17, 0.09))	0.555	-0.70 (-1.91, 0.50)	0.249
	*P* for interaction		0.331		0.231		0.065
	Male	-0.09 (-0.18, 0.001)	0.052	-0.08 (-0.18, 0.03)	0.144	-1.28 (-2.21, -0.35)	0.007
	Female	-0.05 (-0.18, 0.08)	0.460	-0.08 (-0.23, 0.08))	0.326	0.21 (-1.25, 1.67)	0.776

^a^ PM_2.5_, fine particulate matter with aerodynamic diameter < 2.5 μm; PM_10_, inhalable particulate matter with aerodynamic diameter < 10 μm; SO_2_, sulfur dioxide; NO_2_, nitrogen dioxide; CO, carbon monoxide; O_3_, ozone; 95% CI, 95% confidence interval

^b^ Model adjusted for age group (< 65 years and ≥ 65 years), sex, body mass index, smoking status, temperature, season (warm: April to September; cold: October to March), cardiovascular disease, hypertension, diabetes mellitus, resting heart rate, and low-density lipoprotein cholesterol. The long-term exposure model did not adjust for temperature and season

In sensitivity analyses using Model 1, the associations between short- and long-term exposure to air pollution and FMD after adjusting for the year of observation are shown in the Additional file: Table S5. Short-term exposure to ozone and long-term exposure to PM_10_ and NO_2_ were still strongly associated with endothelial dysfunction after adjusting for the year of observation. Other results were close to those of Model 1 in Tables 2 and 3. In sensitivity analyses combining both short- and long-term exposures (Additional file: Table S6), we observed that the effects of mainly four gaseous pollutants could co-exist or tend to be significant. It was worth noting that long-term exposure to air pollutants had higher effect values than short-term exposure. Finally, we developed 2-pollutant models to observe the effects of different pollutants. The analysis of the two-pollutant models based on Model 1 is presented in the Additional file: Table S7 and Table S8. The results of the two-pollutant model for long-term exposure to air pollutants other than ozone were not reported because of too high correlations. We found that 7-day exposure to ozone was significantly associated with lower FMD in all 2-pollutant models. Compared with the single-pollutant models, larger adverse effects were observed for each 10 µg/m^3^ increase in long-term exposure concentrations of NO_2_.

## Discussion

In this study, we demonstrated that long-term exposure to higher concentrations of all air pollutants was significantly associated with more severe endothelial dysfunction. 7-day exposure to PM_2.5_ and PM_10_ was also linearly associated with impaired FMD, whereas ozone had nonlinear associations. Notably, when stratified by age and sex, these apparent adverse associations persisted only among men and those younger than 65 years. To the best of our knowledge, this is the first study to investigate the association of short- and long-term exposure to six air pollutants with endothelial dysfunction in different sex and age subgroups in a highly polluted area, not only demonstrating the age- and sex-specific differences and nonlinearity in the above associations, but also identifying high-risk populations and providing evidence to improve prevention strategies.

Our findings were supported by previous studies and explored the obvious associations between more kinds of pollutants and impaired FMD. A study of 615 clinically healthy participants found that each 1 µg/m^3^ increase in 12-month moving-average estimates of PM_2.5_ and PM_10_ exposure was associated with a 0.07% and 0.09% decrease in FMD [14]. Another study based on a large population found that each 3 µg/m^3^ increase in long-term PM_2.5_ concentration was associated with a 0.3% reduction in FMD, and younger participants seemed to show a greater association [13]. The Framingham cohort study confirmed that a 1.99 µg/m^3^ difference in PM_2.5_ was associated with − 0.16% lower FMD [16]. The only study conducted in China assessed the effect of PM_2.5_ on FMD and obtained similar results [15]. There were no studies providing evidence of the effects of long-term exposure to gaseous pollutants on endothelial dysfunction. Thus, our study greatly remedies the lack of current research on gaseous pollutants and noticed that the effect of NO_2_ was even higher than that of particulate matter. More importantly, we visualized associations between long-term exposure to pollutants and FMD through restricted cubic splines. The lack of positive results from previous studies might be explained due to the low level of air pollutants. Biased exposure estimates due to active reduction in outdoor activities during heavy pollution might be an additional important confounding factor [20]. Compared to previous studies, our study included more participants with complex clinical conditions and assessed more air pollutants and wider exposure levels, which greatly extended the applicability of the findings. Considering that our multi-year study used the same monitoring stations to assign exposures to participants in a smaller region, the long-term exposures of the participants may have involved different temporal rather than spatial contrasts. We obtained similar results after controlling for potential temporal trends and still observed significant associations of NO_2_ and PM_10_ with endothelial dysfunction, demonstrating the reliability of the results. This suggests that adjusting for the year of observation did not affect the associations materially.

After stratification, we found that chronic exposure to air pollution was associated with impaired FMD in men and younger participants only. The study of Amish participants found that the association between particulate matter and FMD was significant and stronger only in men [14]. In particular, only in the subgroup of men younger than 50 years old did the association between PM_10_ and FMD reach statistical significance. Possible explanations were that men engaged in more outdoor activities than women, which led to potential higher exposure in practical daily life. The protective hormonal effects and lower smoking rates in women were also known to improve the clinical status of patients [21, 22]. Besides, younger participants and male participants in our study had higher BMI, and obesity was found to exacerbate the adverse effects of air pollution [23, 24]. Obesity-induced inflammation and metabolic abnormalities might lead to increased inflammatory factors and vascular endothelial growth factors (VEGFs), which could impair vascular function [25, 26]. Meanwhile, the higher proportion of medication use due to comorbidities in older people was also a considerable influence, especially as angiotensin-converting enzyme inhibitors (ACEIs) have been demonstrated to improve endothelial function [27, 28]. In addition, pre-existing endothelial dysfunction in older people might make the effects of air pollution imperceptible. The underlying mechanisms of differences in effects remain to be explored. Nonetheless, as we work towards increasing protection for high-risk groups in the future, we must also pay simultaneous attention to the health risks of air pollution for the older population.

For short-term exposures, we observed significant associations of endothelial dysfunction with 7-day exposure to particulate matter rather than 14 days. Epidemiological studies on the effects of short-term exposure on endothelial function have yielded inconsistent results. The large cohort study found no association between PM_2.5_ on the day before the examination and FMD [13]. Another study of 40 healthy male nonsmokers found that 5-day exposure to SO_2_ and NO_2_ significantly reduced FMD, but not particulate matter [29]. It was concluded that gaseous pollutants affected the endothelial function of the large artery, while particulate matter mainly enlarged the arteriolar dilation response to ischemia. The study of pregnant women found that 14-day exposure to PM_10_ but not PM_2.5_ significantly increased levels of soluble vascular cell adhesion molecule 1 (sVCAM-1), a marker of endothelial dysfunction, providing evidence of a mechanism [30]. Excessively long time-window may be responsible for weakening the association between pollutants and FMD when assessing short-term exposure effects. However, we should be equally concerned about the adverse effects of 14-day exposure to ozone on endothelial dysfunction.

In our subgroup analysis, the associations between short-term exposure and FMD were also only seen in men and participants younger than 65 years. The significant interaction effect showed men were more sensitive to the adverse effects of SO_2_. Similarly, population characteristics such as being young, male, and overweight might account for the increased vulnerability to short-term exposure to air pollution, which was supported by previous meta-analyses from low-pollution areas [31]. Despite the higher prevalence of CVD and hypertension in the older subgroup, as with the results of long-term exposure, the traditionally perceived higher susceptibility was also not observed. The use of anti-hypertensive and vasoactive drugs might help to resist the effects of short-term exposure to air pollution more prominently [32].

Several trials have attempted to elucidate possible pathophysiological mechanisms of air pollutants on endothelial function. In healthy subjects, acute exposure to diesel exhaust could impair NO-mediated endothelial vasomotor function and promote the production of reactive oxygen species (ROS) in endothelial cells to increase oxidative stress [33]. Besides, inhalation of diluted diesel exhaust could also impair the regulation of vascular tone and endogenous fibrinolysis [34]. Promoting inflammation by increasing pro-inflammatory cytokines and endothelins is another harmful mechanism of air pollutants in the proximity of the steel mill site [35]. Although ozone exposure has been shown to impair endothelial function in animal studies, the evidence of its cardiovascular effects in humans is still limited [36].

## Limitations

Firstly, the unavailability of data such as ultrafine particles, traffic noise, dust, indoor time, and cooking fuel use might lead to biased estimates of air pollution exposure. Secondly, there may be potential unidentifiable confounders that are unable to be adjusted for, which is a common shortcoming of observational studies. In addition, due to the lack of relevant data, we could not rule out possible influences of socio-economic or other characteristic (e.g., occupation, household income, and education level) variables.

## Conclusions

This study reveals significant age and sex differences in endothelial dysfunction associated with short- and long-term exposure to different air pollutants over a wider range of concentrations. Further identification of different susceptible populations and proactive measures to mitigate air pollution may be beneficial in reducing the burden of CVD.

### Electronic supplementary material

Below is the link to the electronic supplementary material.

Supplementary Material 1

## Data Availability

The datasets used and/or analyzed during the current study are available from the corresponding author on reasonable request.

## Abbreviations

PM_2.5_: Fine particulate matter with aerodynamic diameter < 2.5 μm
PM_10_: Inhalable particulate matter with aerodynamic diameter < 10 μm
SO_2_: Sulfur dioxide
NO_2_: Nitrogen dioxide
CO: Carbon monoxide
O_3_: Ozone
FMD: Flow-mediated dilation
