# Supplementary material for: Age and sex differences in the effects of short- and long-term exposure to air pollution on endothelial dysfunction
Source: Environ Health. 2024 Jul 8;23:63. doi: 10.1186/s12940-024-01100-3 (PMC11229304; doi:10.1186/s12940-024-01100-3)
Supplement: Supplementary file 1 — Supplementary Material 1 [file 12940_2024_1100_MOESM1_ESM.docx]

**Supplementary Materials**

Table S1. Summary statistics of average exposure concentrations of air pollutants and average temperature for all participants.^a^

|  |  | Minimum | Percentile | | | Maximum |
| --- | --- | --- | --- | --- | --- | --- |
|  |  |  | 25th | 50th | 75th |  |
| PM_2.5_, μg/m^3^ | 7-day | 13.1 | 31.0 | 43.1 | 62.1 | 294.7 |
|  | 14-day | 17.7 | 34.7 | 44.6 | 58.1 | 199.1 |
|  | 12-month | 40.7 | 46.3 | 49.7 | 64.9 | 79.2 |
| PM_10_, μg/m^3^ | 7-day | 26.9 | 50.6 | 68.4 | 90.8 | 323.3 |
|  | 14-day | 32.4 | 53.9 | 68.2 | 91.7 | 214.9 |
|  | 12-month | 66.8 | 73.3 | 78.8 | 93.2 | 103.5 |
| SO_2_, μg/m^3^ | 7-day | 2.1 | 3.0 | 4.1 | 6.7 | 31.6 |
|  | 14-day | 2.2 | 3.1 | 4.1 | 6.8 | 29.1 |
|  | 12-month | 4.3 | 4.9 | 5.5 | 8.4 | 10.5 |
| NO_2_, μg/m^3^ | 7-day | 18.7 | 30.3 | 39.0 | 49.4 | 127.6 |
|  | 14-day | 21.4 | 30.5 | 38.8 | 47.9 | 97.8 |
|  | 12-month | 36.2 | 38.2 | 39.5 | 46.8 | 50.8 |
| CO, mg/m^3^ | 7-day | 0.4 | 0.6 | 0.7 | 0.9 | 5.3 |
|  | 14-day | 0.4 | 0.6 | 0.8 | 0.9 | 3.7 |
|  | 12-month | 0.7 | 0.7 | 0.8 | 1.1 | 1.3 |
| O_3_, μg/m^3^ | 7-day | 2.4 | 42.9 | 88.4 | 133.7 | 242.3 |
|  | 14-day | 2.5 | 42.9 | 87.5 | 132.7 | 215.6 |
|  | 12-month | 91.1 | 93.2 | 95.5 | 97.3 | 100.7 |
| Temperature, ℃ | 7-day | -7.9 | 3.1 | 14.2 | 25.0 | 31.7 |
|  | 14-day | -6.1 | 3.7 | 15.0 | 25.2 | 30.2 |

^a^ PM_2.5_, fine particulate matter with aerodynamic diameter <2.5 μm; PM_10_, inhalable particulate matter with aerodynamic diameter <10 μm; SO_2,_ sulfur dioxide; NO_2,_ nitrogen dioxide; CO, carbon monoxide; O_3_, ozone.

Table S2. Spearman's correlation analysis between 7-day exposure levels of different air pollutants.^a^

|  | PM_2.5_ | PM_10_ | SO_2_ | NO_2_ | CO | O_3_ |
| --- | --- | --- | --- | --- | --- | --- |
| PM_2.5_ | - | 0.808^*^ | 0.428^*^ | 0.612^*^ | 0.755^*^ | -0.157^*^ |
| PM_10_ | 0.808^*^ | - | 0.599^*^ | 0.651^*^ | 0.508^*^ | -0.172^*^ |
| SO_2_ | 0.428^*^ | 0.599^*^ | - | 0.618^*^ | 0.488^*^ | -0.448^*^ |
| NO_2_ | 0.612^*^ | 0.651^*^ | 0.618^*^ | - | 0.633^*^ | -0.645^*^ |
| CO | 0.755^*^ | 0.508^*^ | 0.488^*^ | 0.633^*^ | - | -0.373^*^ |
| O_3_ | -0.157^*^ | -0.172^*^ | -0.448^*^ | -0.645^*^ | -0.373^*^ | - |

^a^ PM_2.5_, fine particles with aerodynamic diameter <2.5 μm; PM_10_, inhalable particles with aerodynamic diameter <10 μm; SO_2_, sulfur dioxide; NO_2_, nitrogen dioxide; CO, carbon monoxide; O_3_, ozone.

^*^ *P* value <0.01.

Table S3. Spearman's correlation analysis between 14-day exposure levels of different air pollutants.^a^

|  | PM_2.5_ | PM_10_ | SO_2_ | NO_2_ | CO | O_3_ |
| --- | --- | --- | --- | --- | --- | --- |
| PM_2.5_ | - | 0.793^*^ | 0.473^*^ | 0.619^*^ | 0.696^*^ | -0.238^*^ |
| PM_10_ | 0.793^*^ | - | 0.656^*^ | 0.647^*^ | 0.439^*^ | -0.256^*^ |
| SO_2_ | 0.473^*^ | 0.656^*^ | - | 0.650^*^ | 0.483^*^ | -0.517^*^ |
| NO_2_ | 0.619^*^ | 0.647^*^ | 0.650^*^ | - | 0.616^*^ | -0.720^*^ |
| CO | 0.696^*^ | 0.439^*^ | 0.483^*^ | 0.616^*^ | - | -0.444^*^ |
| O_3_ | -0.238^*^ | -0.256^*^ | -0.517^*^ | -0.720^*^ | -0.444^*^ | - |

^a^ PM_2.5_, fine particles with aerodynamic diameter <2.5 μm; PM_10_, inhalable particles with aerodynamic diameter <10 μm; SO_2_, sulfur dioxide; NO_2_, nitrogen dioxide; CO, carbon monoxide; O_3_, ozone.

^*^ *P* value <0.01.

Table S4. Spearman's correlation analysis between 12-month exposure levels of different air pollutants.^a^

|  | PM_2.5_ | PM_10_ | SO_2_ | NO_2_ | CO | O_3_ |
| --- | --- | --- | --- | --- | --- | --- |
| PM_2.5_ | - | 0.978^*^ | 0.936^*^ | 0.968^*^ | 0.979^*^ | 0.287^*^ |
| PM_10_ | 0.978^*^ | - | 0.922^*^ | 0.977^*^ | 0.957^*^ | 0.334^*^ |
| SO_2_ | 0.936^*^ | 0.922^*^ | - | 0.915^*^ | 0.937^*^ | 0.297^*^ |
| NO_2_ | 0.968^*^ | 0.977^*^ | 0.915^*^ | - | 0.944^*^ | 0.331^*^ |
| CO | 0.979^*^ | 0.957^*^ | 0.937^*^ | 0.944^*^ | - | 0.288^*^ |
| O_3_ | 0.287^*^ | 0.334^*^ | 0.297^*^ | 0.331^*^ | 0.288^*^ | - |

^a^ PM_2.5_, fine particles with aerodynamic diameter <2.5 μm; PM_10_, inhalable particles with aerodynamic diameter <10 μm; SO_2_, sulfur dioxide; NO_2_, nitrogen dioxide; CO, carbon monoxide; O_3_, ozone.

^*^ *P* value <0.01.

Table S5. Associations between exposure to air pollution and flow-mediated dilation (%) after adjusting for the year of observation.^a,b^

|  | 7-day | | 14-day | | 12-month | |
| --- | --- | --- | --- | --- | --- | --- |
|  | *β* (95% CI) | *P* value | *β* (95% CI) | *P* value | *β* (95% CI) | *P* value |
| PM_2.5_, per 10 μg/m^3^ | -0.04 (-0.11, 0.03) | 0.281 | 0.04 (-0.06, 0.13) | 0.453 | -0.21 (-0.57, 0.16) | 0.267 |
| PM_10_, per 10 μg/m^3^ | -0.04 (-0.09, 0.02) | 0.202 | 0.001 (-0.07, 0.07) | 0.981 | -0.38 (-0.74, -0.008) | 0.045 |
| SO_2_, per 1 μg/m^3^ | 0.01 (-0.05, 0.07) | 0.760 | 0.03 (-0.05, 0.10) | 0.479 | -0.19 (-0.44, 0.06) | 0.127 |
| NO_2_, per 10 μg/m^3^ | 0.05 (-0.13, 0.23) | 0.593 | 0.21 (-0.03, 0.46) | 0.083 | -1.20 (-1.98, -0.42) | 0.003 |
| CO, per 0.1 mg/m^3^ | -0.001 (-0.05, 0.05) | 0.966 | 0.06 (-0.003, 0.13) | 0.063 | -0.16 (-0.40, 0.07) | 0.168 |
| O_3_, per 10 μg/m^3^ | -0.09 (-0.16, -0.01) | 0.026 | -0.10 (-0.19, -0.009) | 0.031 | -0.72 (-1.56, 0.13) | 0.096 |

^a^ PM_2.5_, fine particulate matter with aerodynamic diameter <2.5 μm; PM_10_, inhalable particulate matter with aerodynamic diameter <10 μm; SO_2_, sulfur dioxide; NO_2_, nitrogen dioxide; CO, carbon monoxide; O_3_, ozone; 95% CI, 95% confidence interval.

^b^ Model adjusted for age group (<65 years and ≥65 years), sex, body mass index, smoking status, temperature, season (warm: April to September; cold: October to March), and the year of observation. The long-term exposure model did not adjust for temperature and season.

Table S6. Associations between the combination of short- and long-term exposure to air pollution and flow-mediated dilation (%).^a,b^

| Combination of short- and long-term exposure | 7-day | | 12-month | |  | 14-day | | 12-month |  |
| --- | --- | --- | --- | --- | --- | --- | --- | --- | --- |
|  | *β* (95% CI) | *P* value | *β* (95% CI) | *P* value |  | *β* (95% CI) | *P* value | *β* (95% CI) | *P* value |
| PM_2.5_, per 10 μg/m^3^ | -0.04 (-0.11, 0.04) | 0.347 | -0.08 (-0.25, 0.10) | 0.398 |  | 0.05 (-0.05, 0.14) | 0.368 | -0.15 (-0.33, 0.03) | 0.107 |
| PM_10_, per 10 μg/m^3^ | -0.03 (-0.08, 0.03) | 0.315 | -0.12 (-0.31, 0.06) | 0.200 |  | 0.01 (-0.06, 0.09) | 0.750 | -0.16 (-0.36, 0.03) | 0.092 |
| SO_2_, per 1 μg/m^3^ | 0.02 (-0.05, 0.09) | 0.550 | -0.09 (-0.20, 0.02) | 0.116 |  | 0.04 (-0.04, 0.12) | 0.312 | -0.10 (-0.21, 0.01) | 0.077 |
| NO_2_, per 10 μg/m^3^ | 0.08 (-0.10, 0.26) | 0.384 | -0.56 (-1.00, -0.12) | 0.012 |  | 0.24 (0.009, 0.46) | 0.041 | -0.68 (-1.13, -0.23) | 0.003 |
| CO, per 0.1 mg/m^3^ | 0.004 (-0.05, 0.05) | 0.871 | -0.07 (-0.18, 0.03) | 0.170 |  | 0.07 (0.004, 0.13) | 0.036 | -0.13 (-0.24, -0.02) | 0.019 |
| O_3_, per 10 μg/m^3^ | -0.08 (-0.15, 0.00) | 0.050 | -0.70 (-1.51, 0.10) | 0.086 |  | -0.09 (-0.17, 0.002) | 0.055 | -0.73 (-1.53, 0.07) | 0.074 |

^a^ PM_2.5_, fine particulate matter with aerodynamic diameter <2.5 μm; PM_10_, inhalable particulate matter with aerodynamic diameter <10 μm; SO_2_, sulfur dioxide; NO_2_, nitrogen dioxide; CO, carbon monoxide; O_3_, ozone; 95% CI, 95% confidence interval.

^b^ Model adjusted for age group (<65 years and ≥65 years), sex, body mass index, smoking status, temperature, and season (warm: April to September; cold: October to March).

Table S7. Associations between short-term exposure to air pollution and flow-mediated dilation (%) using 2-pollutant models.^a,b^

| 2-pollutant model | | 7-day | | 14-day | |
| --- | --- | --- | --- | --- | --- |
|  |  | *β* (95% CI) | *P* value | *β* (95% CI) | *P* value |
| PM_2.5_, per 10 μg/m^3^ | + PM_10_ | -0.01 (-0.13, 0.11) | 0.825 | 0.06 (-0.09, 0.21) | 0.442 |
|  | + SO_2_ | -0.06 (-0.14, 0.02) | 0.117 | 0.001 (-0.10, 0.10) | 0.979 |
|  | + NO_2_ | -0.13 (-0.23, -0.02) | 0.020 | -0.06 (-0.20, 0.07) | 0.364 |
|  | + CO | -0.12 (-0.25, 0.005) | 0.060 | -0.10 (-0.26, 0.05) | 0.193 |
|  | + O_3_ | -0.05 (-0.11, 0.02) | 0.178 | 0.006 (-0.08, 0.09) | 0.888 |
| PM_10_, per 10 μg/m^3^ | + PM_2.5_ | -0.03 (-0.13, 0.06) | 0.502 | -0.05 (-0.17, 0.07) | 0.384 |
|  | + SO_2_ | -0.05 (-0.11, 0.008) | 0.092 | -0.02 (-0.10, 0.05) | 0.590 |
|  | + NO_2_ | -0.09 (-0.16, -0.01) | 0.023 | -0.07 (-0.17, 0.02) | 0.131 |
|  | + CO | -0.05 (-0.12, 0.01) | 0.113 | -0.05 (-0.13, 0.03) | 0.233 |
|  | + O_3_ | -0.04 (-0.09, 0.01) | 0.157 | -0.01 (-0.08, 0.06) | 0.733 |
| SO_2_, per 1 μg/m^3^ | + PM_2.5_ | 0.02 (-0.05, 0.09) | 0.494 | 0.004 (-0.07, 0.08) | 0.911 |
|  | + PM_10_ | 0.02 (-0.04, 0.09) | 0.506 | 0.01 (-0.06, 0.09) | 0.712 |
|  | + NO_2_ | -0.006 (-0.07, 0.06) | 0.861 | -0.007 (-0.08, 0.06) | 0.838 |
|  | + CO | 0.006 (-0.07, 0.08) | 0.866 | -0.02 (-0.11, 0.06) | 0.592 |
|  | + O_3_ | 0.01 (-0.05, 0.08) | 0.670 | 0.03 (-0.04, 0.10) | 0.465 |
| NO_2_, per 10 μg/m^3^ | + PM_2.5_ | 0.25 (-0.02, 0.52) | 0.065 | 0.21 (-0.12, 0.53) | 0.206 |
|  | + PM_10_ | 0.20 (-0.04, 0.44) | 0.098 | 0.26 (-0.04, 0.55) | 0.089 |
|  | + SO_2_ | 0.01 (-0.18, 0.20) | 0.913 | 0.10 (-0.12, 0.33) | 0.370 |
|  | + CO | 0.08 (-0.17, 0.33) | 0.538 | 0.04 (-0.25, 0.33) | 0.782 |
|  | + O_3_ | -0.03 (-0.20, 0.14) | 0.763 | 0.05 (-0.16, 0.26) | 0.641 |
| CO, per 0.1 mg/m^3^ | + PM_2.5_ | 0.06 (-0.03, 0.14) | 0.183 | 0.08 (-0.02, 0.18) | 0.104 |
|  | + PM_10_ | 0.02 (-0.04, 0.07) | 0.555 | 0.05 (-0.02, 0.12) | 0.137 |
|  | + SO_2_ | -0.01 (-0.07, 0.04) | 0.602 | 0.04 (-0.03, 0.11) | 0.267 |
|  | + NO_2_ | -0.03 (-0.09, 0.04) | 0.423 | 0.02 (-0.06, 0.10) | 0.611 |
|  | + O_3_ | -0.01 (-0.06, 0.03) | 0.553 | 0.03 (-0.03, 0.08) | 0.377 |
| O_3_, per 10 μg/m^3^ | + PM_2.5_ | -0.08 (-0.15, -0.003) | 0.042 | -0.09 (-0.18, -0.001) | 0.047 |
|  | + PM_10_ | -0.08 (-0.15, -0.001) | 0.048 | -0.09 (-0.18, -0.00) | 0.049 |
|  | + SO_2_ | -0.08 (-0.16, -0.006) | 0.034 | -0.10 (-0.19, -0.007) | 0.035 |
|  | + NO_2_ | -0.08 (-0.16, -0.006) | 0.035 | -0.08 (-0.17, 0.006) | 0.066 |
|  | + CO | -0.08 (-0.16, -0.005) | 0.035 | -0.09 (-0.17, 0.001) | 0.053 |

^a^ PM_2.5_, fine particulate matter with aerodynamic diameter <2.5 μm; PM_10_, inhalable particulate matter with aerodynamic diameter <10 μm; SO_2_, sulfur dioxide; NO_2_, nitrogen dioxide; CO, carbon monoxide; O_3_, ozone; 95% CI, 95% confidence interval.

^b^ Model adjusted for age group (<65 years and ≥65 years), sex, body mass index, smoking status, temperature, and season (warm: April to September; cold: October to March).

Table S8. Associations between long-term exposure to air pollution and flow-mediated dilation (%) using 2-pollutant models.^a,b^

| 2-pollutant model | | 12-month | |
| --- | --- | --- | --- |
|  |  | *β* (95% CI) | *P* value |
| PM_2.5_, per 10 μg/m^3^ | + O_3_ | -0.07 (-0.24, 0.09) | 0.396 |
| PM_10_, per 10 μg/m^3^ | + O_3_ | -0.12 (-0.30, 0.07) | 0.206 |
| SO_2_, per 1 μg/m^3^ | + O_3_ | -0.05 (-0.15, 0.06) | 0.379 |
| NO_2_, per 10 μg/m^3^ | + O_3_ | -0.42 (-0.87, 0.03) | 0.067 |
| CO, per 0.1 mg/m^3^ | + O_3_ | -0.04 (-0.14, 0.06) | 0.388 |
| O_3_, per 10 μg/m^3^ | + PM_2.5_ | -0.66 (-1.51, 0.18) | 0.123 |
|  | + PM_10_ | -0.59 (-1.44, 0.26) | 0.174 |
|  | + SO_2_ | -0.63 (-1.49, 0.23) | 0.152 |
|  | + NO_2_ | -0.45 (-1.32, 0.43) | 0.317 |
|  | + CO | -0.64 (-1.50, 0.21) | 0.142 |

^a^ PM_2.5_, fine particulate matter with aerodynamic diameter <2.5 μm; PM_10_, inhalable particulate matter with aerodynamic diameter <10 μm; SO_2_, sulfur dioxide; NO_2_, nitrogen dioxide; CO, carbon monoxide; O_3_, ozone; 95% CI, 95% confidence interval.

^b^ Model adjusted for age group (<65 years and ≥65 years), sex, body mass index, and smoking status. The other results for air pollutants were not reported because of too high correlations.


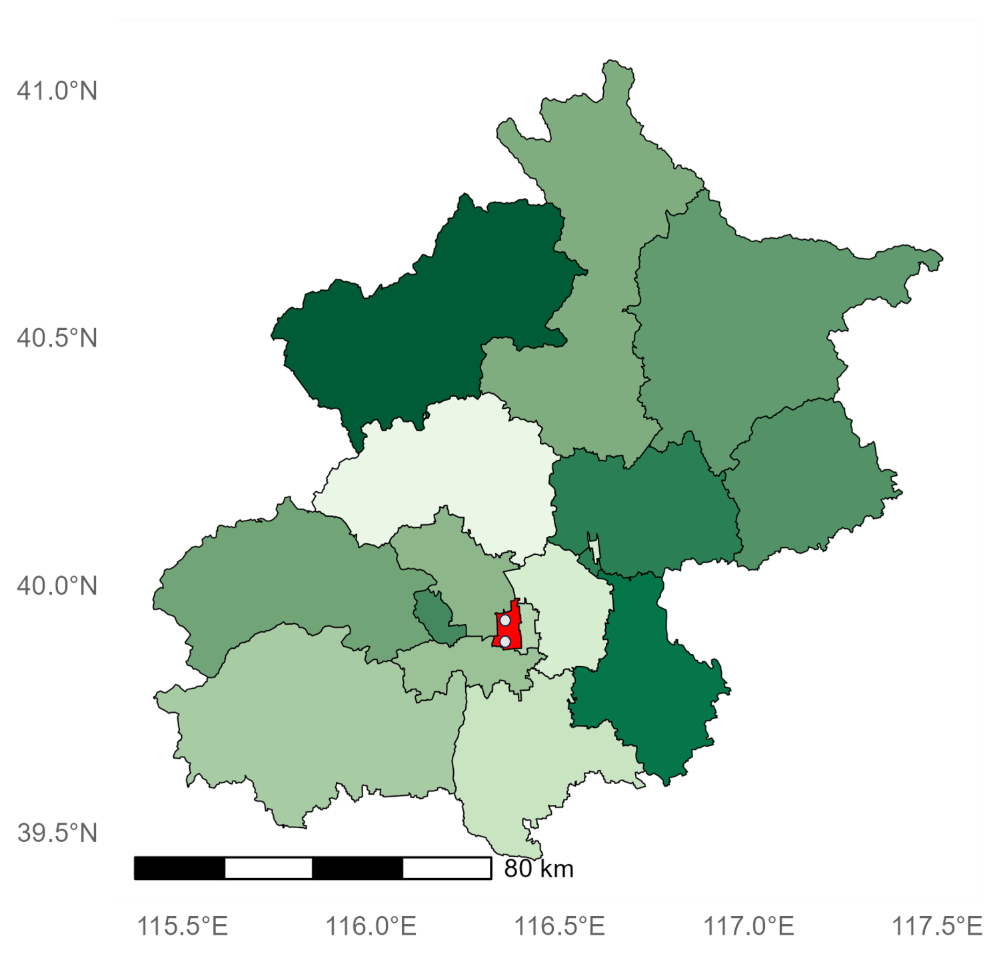


Figure S1. Distribution of two fixed ambient air quality monitoring stations in Xicheng District, Beijing.^a^

^a^ The white spots are the locations of two fixed air monitoring stations. The geographic extent of Xicheng District in Beijing is highlighted in red.
